# Cerebral Venous Sinus Thrombosis Associated With Subclinical Hypothyroidism: A Case Report and Literature Review

**DOI:** 10.7759/cureus.62333

**Published:** 2024-06-13

**Authors:** Takuya Wakabayashi, Shigeki Takada, Yoshitaka Tsujimoto, Nobutake Sadamasa, Waro Taki

**Affiliations:** 1 Department of Neurosurgery, Stroke Center, Koseikai Takeda Hospital, Kyoto, JPN

**Keywords:** anticoagulation therapy, thyroid dysfunction, thyroid-stimulating hormone (tsh), subclinical hypothyroidism, cerebral venous sinus thrombosis (cvst)

## Abstract

Thyroid dysfunction is a well-known cause of cerebral venous sinus thrombosis (CVST), but most reports have focused on CVST associated with hyperthyroidism, with only a few mentioning CVST associated with hypothyroidism. Subclinical hypothyroidism, characterized by thyroid hormone levels within reference values but elevated thyroid-stimulating hormone, can also cause CVST. Here, we present a case of CVST associated with subclinical hypothyroidism. A 48-year-old man with headache, nausea, and left-sided motor weakness was admitted to our hospital, with a history of economy-class syndrome. Magnetic resonance imaging revealed occlusion of the superior sagittal sinus, right transverse sinus, and right sigmoid sinus. Digital subtraction angiography (DSA) confirmed CVST from the right common carotid artery, revealing abnormal staining of the thyroid gland. The patient was serologically in a state of subclinical hypothyroidism. Consequently, the patient was diagnosed with CVST associated with subclinical hypothyroidism. Anticoagulation therapy was initiated shortly after admission. CVST gradually resolved, and the affected sinuses were recanalized. Paraplegia improved, and the patient was discharged home 19 days after admission with a modified Rankin scale of 1. Subclinical hypothyroidism can induce CVST, underscoring the importance of screening for thyroid function in CVST patients, even without apparent thyroid dysfunction symptoms. DSA findings are valuable for diagnosing thyroid disease.

## Introduction

Thyroid dysfunction can be a cause of cerebral venous sinus thrombosis (CVST), but most studies demonstrated that CVST is associated with hyperthyroidism. However, there are also several reports of CVST related to hypothyroidism [1-6].

Hypothyroidism can be classified into overt hypothyroidism and subclinical hypothyroidism. Subclinical hypothyroidism is a condition in which thyroid hormone levels are maintained, but thyroid-stimulating hormone (TSH) levels are elevated. CVST associated with subclinical hypothyroidism is rare. Herein, we describe a case of CSVT that was thought to be caused by subclinical hypothyroidism, with a review of the literature.

## Case presentation

**Figure 1:**
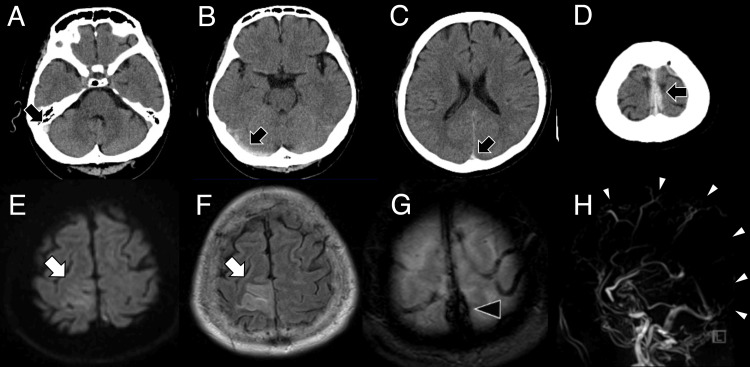
Head CT and MRI at the onset Head CT showed high-density areas (black arrows) in the SSS, right TS, and SS (A–D). Head MRI showed hyperintensity areas (white arrows) in DWI (E) and FLAIR (F). T2*-WI showed a low signal (black arrowhead) in the SSS (G), and MRV did not depict the SSS (white arrowheads) (H). CT = computed tomography; DWI = diffusion-weighted imaging; FLAIR = Fluid attenuated inversion recovery; MRI = magnetic resonance imaging; MRV = magnetic resonance venography; SS = sigmoid sinus; SSS = superior sagittal sinus; TS = transverse sinus; T2*-WI = T2*-weighted image

**Figure 2:**
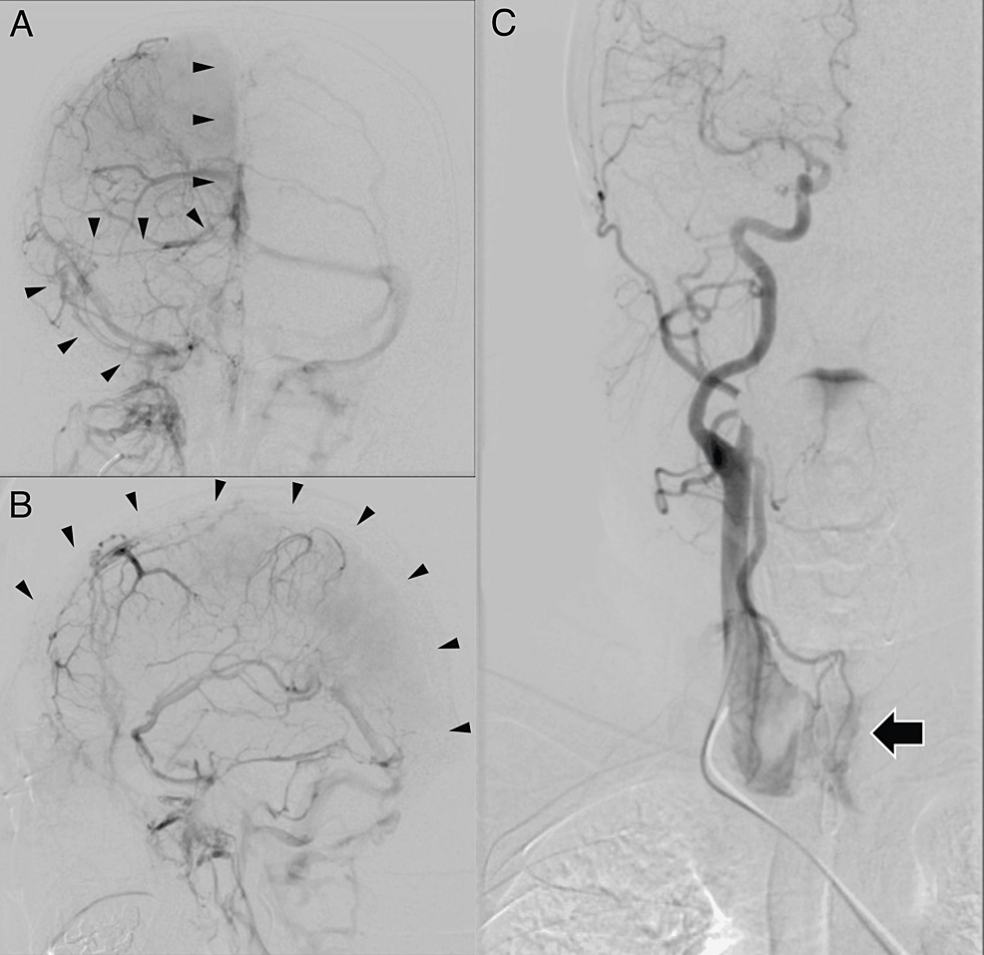
Cerebral angiography demonstrating CVST and abnormal staining in the thyroid gland Right ICAG showed CVST (black arrowheads) from SSS to right SS (A and B). Right CCAG showed abnormal staining in the thyroid gland (black arrow) (C). CCAG = common carotid artery angiography; CVST = cerebral venous sinus thrombosis; ICAG = internal carotid artery angiography; SS = sigmoid sinus; SSS = superior sagittal sinus

A 48-year-old male, with a history of economy class syndrome (deep vein thrombosis in the leg) and hyperthyroidism, was admitted to our hospital with headache, vomiting, and left hemiparesis (the modified Rankin scale at the time of admission was 2). Head computed tomography (CT) showed high-density areas in the superior sagittal sinus (SSS), right transverse sinus (TS), and sigmoid sinus (SS, Figures 1A-1D). Magnetic resonance imaging (MRI) revealed a hyperintensity area in the right frontal lobe on diffusion-weighted imaging (DWI) and fluid-attenuated inversion recovery (FLAIR) (Figures 1E, 1F). A T2*-weighted image demonstrated hypointensity in the SSS and the cortical vein, while magnetic resonance venography (MRV) showed a lack of flow in the SSS (Figures 1G, 1H). Digital subtraction angiography (DSA) confirmed occlusion of the SSS, the right TS, and the right SS (Figures 2A, 2B), leading to a diagnosis of CVST. Continuous heparin infusion commenced shortly after diagnosis. Plasminogen activator inhibitor-1 (PAI-1) (42.4 ng/mL; reference values: 0-50.0 ng/mL), homocysteine (8.8 μmol/L; reference values: 6.3-18.9 μmol/L), protein C plasma level (116%; reference values: 70-140%), and protein S plasma level (136%; reference values: 70-140%) were normal. Right common carotid artery angiography (CCAG) revealed abnormal staining in the thyroid gland (Figure 2C). Despite normal free T3 (2.59 pg/mL; reference values: 1.68-3.67 pg/mL) and free T4 (2.56 pg/mL; reference values: 0.7-1.48 pg/mL), TSH (6.39 μIU/mL; reference values: 0.35-4.94 μIU/mL) was elevated, leading to a diagnosis of CVST associated with subclinical hypothyroidism. The diagnosis of chronic thyroiditis was serologically confirmed based on negative thyroid-stimulating antibodies, positive thyroid peroxidase antibodies (TPOAb), and thyroglobulin antibodies (TgAb). Thyroid hormone replacement therapy was not initiated for the patient.

MRI showed that CVST was gradually dissolved, and the SSS, TS, and SS were recanalized. Ten days after admission, continuous heparin infusion was switched to warfarin. Nineteen days after admission, he was discharged home with a slight coordinated movement disorder of the left hand (the modified Rankin scale of 1). Two weeks after being discharged, he had no neurological deficit. He continued to take warfarin for one year. Since he stopped taking warfarin, no recurrence of venous thrombosis was noted.

## Discussion

Both hyperthyroidism and hypothyroidism can cause CVST and thrombophilia [7]. However, CVST caused by subclinical hypothyroidism is rare [1-6]. In this case, hypothyroidism may be involved in thrombus formation even in subclinical conditions. Thus, it is important to measure the TSH and thyroid hormone levels.

CVST is generally associated with poor outcomes [8,9]; however, CVST cases associated with subclinical hypothyroidism, including ours, had a good outcome. Fandler-Höfler et al. have described that 17 of 107 patients with CVST were diagnosed with hypothyroidism [4]. Five of 17 (Table 1, #19-23) were diagnosed with hypothyroidism after the onset of CVST, and all had subclinical hypothyroidism, suggesting an association between CVST and subclinical hypothyroidism. Twelve of 17 (Table 1, #7-18) were diagnosed with hypothyroidism before the onset of CVST. After the diagnosis of hypothyroidism, they developed CVST despite hormone replacement therapy, leading to normal TSH, free T3, and free T4 values [4]. Therefore, the incidence of CVST is relatively high in hypothyroidism despite hormone replacement therapy and normalized hormone levels. This suggests that, in the present case, anticoagulation should be considered as a preventive treatment for CVST. Although no thyroid hormone replacement therapy has been administered, since the patient was diagnosed with subclinical hypothyroidism, TSH spontaneously recovered to baseline levels in this patient. Heparin was used at the time of diagnosis, followed by a long-term continuation of warfarin. The patient showed no recurrence for two years.

**Table 1:** Cases of CVST associated with hypothyroidism ACT = anticoagulation therapy; CVST = cerebral venous sinus thrombosis; F = female; FND = focal neurological deficit; HRT = hormone replacement therapy; IJV = internal jugular vein; Lt = left; M = male; mRS = modified Rankin Scale; N/A = not available; rt = right; SS = sigmoid sinus; SSS = superior sagittal sinus; T3 = Tri-iodothyronine; TS = transverse sinus; TSH = thyroid stimulating hormone; UID = unidentified; WF = warfarin; ↑= increased; → = normal level; ↓= decreased; () = reference value; + = administered; − = not administered

Authors & Year	Case	Age/Sex	TSH	T3	T4	Symptoms	Occlusion areas	Outcome	ACT	HRT
Peralta et al., 2008 [6]	#1	52/F	14.38mU/L↑ (0.35-5)	1.49ng/mL→ (0.6-1.81)	0.7ng/dL↓ (0.89-1.8)	Headache, seizure, rt hemiplegia	SSS, rt TS, cortical vein	No symptoms	UID	+ Before the onset of CVST
Peralta et al., 2008 [6]	#2	21/F	10.85mIU/L↑ (0.35–5.50)	→	→	Headache, seizure	SSS, lt TS-SS	No symptoms	UID	+
Chebel et al., 2010 [2]	#3	37/F	6.79mIU/L↑ (0.25-4)	N/A	1.38ng/mL→ (0,8–1,8)	Seizure, rt hemiplegia	SSS, lt TS-SS	mRS0	Heparin, WF	+ Before the onset of CVST
Aggarwal et al., 2013 [1]	#4	45/F	13.92mIU/mL↑	0.94ng/mL→	8.36μg/dL→	Headache, impaired consciousness, quadriplegia, rt tingling	SSS, lt TS	No symptoms	Heparin, WF	−
Shirisha et al., 2016 [5]	#5	45/M	165μIU/mL↑	0.7pg/mL↓	0.15ng/dL↓	Seizure, incoherent conversation, drowsiness	rt TS	No symptoms	−	+
Douma et al., 2022 [3]	#6	10/F	20μIU/mL↑ (0.34-5.6)	N/A	5pmol/L↓ (7.9-14.4)	Headache, seizure, drowsiness	lt TS-IJV, rt TS, vein of Galen	No symptoms	Heparin, Sintrom	+
Fandler-Höfler et al., 2022 [4]	#7-18	N/A	1.35±1.06 mIU/L→	3.38 ± 0.53 pmol/L→	16.48 ± 4.26 pmol/L→	Headache 100%, seizure 33.3%, impaired vision 16.7%, FND 41.7%	TS 83.3% SSS41.7% deep vein 0% cortical vein 33.3%	mRS0	NA	+ Before the onset of CVST
Fandler-Höfler et al., 2022 [4]	#19-23	N/A	5.45±0.73 mIU/L↑	4.57 ± 0.80 pmol/L→	14.88 ± 3.42 pmol/L→	Headache 60%, seizure 40%, impaired vision 20%, FND 20%	TS 100% SSS 40% deep vein 0% cortical vein 40%	Median mRS1 (at discharge)	NA	NA
Present case	#24	48/M	6.39 mIU/L↑ (0.35-4.94)	2.59pg/mL→ (1.68-3.67)	2.56ng/dL→ (0.7-1.48)	Headache, lt hemiplegia	SSS, rt TS-SS	mRS0	Heparin, WF	−

In overt and subclinical hypothyroidism, coagulation is enhanced by reduced fibrinolytic capacity (high levels of α2-antiplasmin and plasminogen activator inhibitor-1) and high C-reactive protein levels. Overt hypothyroidism further enhances coagulation with hyperhomocysteinemia and elevated thrombin-activated fibrinolysis inhibitor levels [4,6,10,11]. Endothelial dysfunction is also observed in the microvasculature of patients with overt and subclinical hypothyroidism, caused by chronic inflammation and impaired nitric oxide utilization, leading to venous stasis [6,10,12,13]. The prevalence of antiendothelial cell antibodies is increased in hypothyroidism, contributing to the development of CVST [14]. However, PAI-1 and homocysteine are not elevated in subclinical hypothyroidism [10].

While MRI, CT, MRV, and CT venography are less invasive and commonly used to diagnose CVST, DSA proves useful in diagnosing CVST and detecting contrast staining in the thyroid gland, facilitating the precise inspection of thyroid dysfunction. Initially, hyperthyroidism was suspected as the cause of CVST due to the increased blood flow to the thyroid gland. However, serologically, the patient was diagnosed with chronic thyroiditis and subclinical hypothyroidism.

Although endovascular treatment for CVST remains controversial, endovascular treatment in patients with severe symptoms or worsening symptoms despite anticoagulation has been effective and results in good outcomes in patients with early diagnosis and treatment [15,16]. Styczen et al. demonstrated that 80%-90% of patients with CVST refractory to standard therapy or severe CVST have a good outcome (mRS ≤ 2) after endovascular treatment [17]. Placing a stent retriever in the occluded venous sinus as an anchor and removing the thrombus by pushing forward a suction catheter and suctioning the thrombus leads to a high success rate of recanalizing the occluded sinus [17,18]. Mechanical thrombectomy can be a salvage treatment for patients with severe CVT refractory to standard medical therapy. In this case, consideration for this treatment would have been given if symptoms had worsened.

## Conclusions

This case highlights the need for evaluating the thyroid function of patients with CVST, even in the absence of clinical symptoms of thyroid disorders, and suggests that DSA may be useful in the diagnosis of CVST and thyroid dysfunction. Considering the development of CVST despite the normalization of hormone levels after replacement therapy for hypothyroidism, anticoagulation therapy should be considered to prevent recurrence. Further studies are also needed to clarify the causal role of hypothyroidism, including subclinical conditions, on CVST.
